# Design and characterisation of casein coated and drug loaded magnetic nanoparticles for theranostic applications†

**DOI:** 10.1039/d4ra02626h

**Published:** 2024-08-20

**Authors:** Christina Wenck, Nils Meier, Eilien Heinrich, Verena Grützner, Frank Wiekhorst, Regina Bleul

**Affiliations:** a Fraunhofer Institute for Microengineering and Microsystems IMM Carl-Zeiss-Str. 18-20 55129 Mainz Germany regina.bleul@imm.fraunhofer.de; b Metrology for Magnetic Nanoparticles, Physikalisch-Technische Bundesanstalt Abbestr. 2-12 10587 Berlin Germany

## Abstract

Theranostic systems enable early cancer diagnostic and treatment. In this work, we prepared Na-caseinate coated magnetic nanoparticles (MNP) to assess their capability as a theranostic system. This system enables monitoring by magnetic particle imaging (MPI), drug delivery and magnetic hyperthermia. MNP were synthesized in a continuous flow, coated with Na-caseinate and enzymatically crosslinked with transglutaminase to increase their colloidal stability and enable drug loading. They were investigated concerning their magnetic behaviour by DC magnetization measurements (DCM), magnetic particle spectroscopy (MPS) and AC-magnetometry to evaluate their suitability for MPI and hyperthermia. Further, their stability in different salt solutions as well as their encapsulation efficiency with a hydrophobic model drug (nile red), cell viability and uptake were investigated. Our results show that the Na-caseinate coating of MNP marginally effects the magnetic behaviour of the MNP with a consistent magnetization saturation *M*_S_ of 109(5) A m^2^ per kg(Fe) for uncoated and casein coated MNP and with a decrease of <15% of *A*_3_*, but only a slight decrease of 2% of *A*_5_/*A*_3_ for Na-caseinate coated MNP. Furthermore, the Na-caseinate coating of MNP increased their salt stability, under unchanged magnetic behaviour. Drug loading (up to ∼75%) and release kinetics such as the delivery into cutaneous squamous cell carcinoma cells (SCL-1) was shown. Our results demonstrate that casein coated MNP are highly promising candidates for theranostic applications in drug delivery, magnetic hyperthermia and magnetic particle imaging.

## Abstract 2

Magnetic protein-coated, drug-loaded nanohybrids have huge potential in future cancer theranostics for real-time monitored drug delivery.

## Graphical abstract

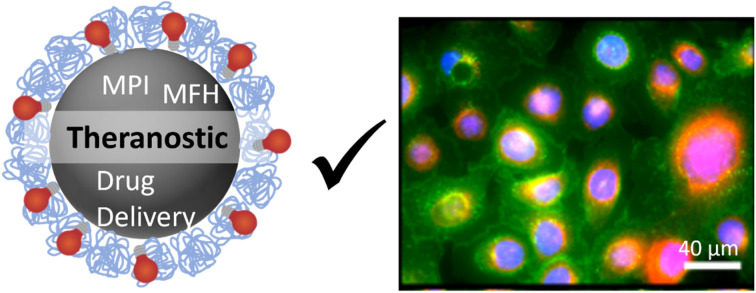

## 1 Introduction

Theranostics (theragnostics) have become a promising and fast-growing research field in nanomedicine in recent years. Theranostic systems are composed of nanoparticles that combine different functionalities for diagnostic imaging and therapeutic intervention (*e.g.*, drug delivery or hyperthermia). Especially in early cancer diagnostic and treatment, theranostic systems are said to possess huge potential to save and improve the life of millions of patients worldwide.^1^

Magnetic nanoparticles (MNP) represent a class of nanoparticles that can be manipulated by magnetic fields, offering theranostic applications. They can be used as local probes in magnetic particle imaging (MPI), magnetic resonance imaging (MRI) or to produce heat in magnetic hyperthermia treatment of cancer. Due to good tolerability and biocompatibility, the commonly used MNP-systems consist of an iron oxide magnetic core with a non-magnetic layer.^1^ They can be produced in a top-down or bottom-up approach, whereby they are either produced from bulk material or by a controlled oxidation of iron, respectively.^2^ Independent from their production procedure, MNP must be stabilized with a coating to protect the surface from oxidation or degradation.^1,2^ Tannic acid is a commonly used stabilizing agent for iron oxide MNP.^3–5^ Still further coatings might be useful to improve the stability in physiological medium.^6^ Protein coatings like bovine serum albumin (BSA) prove to be applicable for this purpose.^7–9^ In a previous study we already showed that BSA coating improved the stability of MNP in a physiologic environment.^6^ A coating with a more functional but still surface-active protein could introduce not only a stabilizing but also a drug-releasing layer. By coating the MNP, simultaneous magnetic particle imaging (MPI) and drug delivery is possible, enabling the MNP to act as a theranostic agent.

Casein, the major protein fraction in mammalian milk, is a natural delivery system for minerals, lipids and amino acids. Casein consists mainly of four different proteins, (α_S1_-, α_S2_-, β- and κ-casein), which assemble to form submicelles. *Via* calcium phosphate bridges the submicelles further aggregate to form casein micelles (∼200 nm). The solubility of casein micelles originates from the κ-casein, located on the micelle surface, which forms soluble Ca^2+^ complexes. The inner part of the micelle (α_S1_-, α_S2_- and β-casein) is mostly hydrophobic due to the nonpolar side chains of the amino acids but also contains water reservoirs.^10,11^

Because of their amphiphilic nature, abundance and ability to form nanostructures, casein and casein micelles have been of great interest for drug delivery applications.^12,13^ In addition, the increase in size of casein with increasing temperature, caused by an increase in the mobility of the individual proteins, could be beneficial for drug delivery applications.^14,15^ Casein was already investigated as a delivery system for anti-cancer drugs such as doxorubicin, daunorubicin and paclitaxel.^16–18^ As such, casein primarily showed no negative side effects: When administered orally, it was shown that the casein micelles themselves would not be absorbed by the digestive system and therefore, there was no indication of any risk.^19^ Direct intravenous injection of casein only caused little immune response.^20^ Casein hydrolysates are a more complex matter, however only mild immune effects, like decrease in the pro-inflammatory cytokines have been found.^21^ These safe attributes, their ability to interact with a huge variety of substances, their self-assembly and temperature as well as pH responsiveness enable casein to be a promising release system.^22^

Studies showed that casein coating of MNP can improve their biocompatibility with a remaining cell viability of >85% (depending on MNP concentration).^23–25^ In addition, promising results for the use of casein-coated MNP for magnetic hyperthermia in *in vivo* performance tests in mice, where hyperthermia reduced tumour size in mice by 33% in 7 days, have been shown.^24^ Several approaches to produce casein coated MNP were already reported.^23–25^ One example is the formation of MNP directly inside of casein micelles, resulting, however, in a wide size distribution and anisotropic nanoparticles.^25^ Another example is the ligand exchange of hydrophobic MNP (coated with oleic acid), which showed higher relaxivity and MRI contrast than the same hydrophobic MNP with a conventional synthetic polymer.^26^ Additionally, a drug delivery system was investigated in which MNP were coated with an inner synthetic polymer and an outer casein layer. This approach allowed drug loading of doxorubicin and indocyanine green into the inner polymer layer, while retaining the ability to be monitored by MRI.^27^

**Fig. 1:**
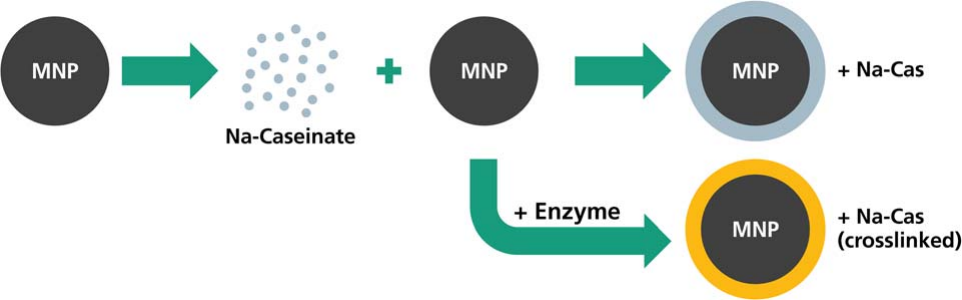
Overview of the prepared and investigated samples and their production procedure.

In this study, we investigated continuously synthesized MNP with casein coating in terms of their magnetic behaviour, colloidal stability and drug encapsulation. MNP were synthesized and then coated with Na-caseinate. In a different setting, the Na-caseinate was further enzymatically crosslinked to potentially increase the stability and drug encapsulation. The different Na-caseinate coating procedures are depicted in Fig. 1. The coated MNP were investigated in terms of particle size, shape and stability before and after casein coating, as well as their magnetic properties (magnetization saturation, MPI and hyperthermia performance). Furthermore, we investigated their suitability as a drug delivery system by studying the encapsulation efficiency and the release of lipophilic model drug (nile red) and their performance in cell viability studies. Here, the magnetic core of the hybrid nanoparticle system can act as a tracer for *in vivo* monitoring by MPI,^28^ providing the diagnostic component and further also enabling its application in magnetic hyperthermia.

The aim of this study was to assess the suitability of casein coated MNP as a theranostic system for application in drug delivery, magnetic hyperthermia and magnetic particle imaging.

## 2 Experimental

### 2.1. Materials

Iron(ii) chloride tetrahydrate (≥99%), nile red (for microscopy), casein sodium salt (from bovine milk), sodium nitrate (Ph. Eur.), sodium hydroxide, sucrose (Ph. Eur.), potassium dihydrogen phosphate (p.a.), Iron(ii,iii) oxide (98%), hydroxylamine hydrochloride (99%) and 1,10-phenanthrolin monohydrate (reagent grade) were purchased from Sigma-Aldrich (Taufkirchen, Germany). Polysorbate 80 (Tween® 80) was purchased from Carl Roth (Karlsruhe, Germany). Potassium chloride (Ph. Eur.), sodium chloride (Ph. Eur.), sodium carbonate (anhydrous, Ph. Eur.), magnesium sulphate (anhydrous, EssentQ) and acetic acid (Ph. Eur.) were bought from Scharlau (Barcelona, Spain). Ammonium sulfate (Reag. Ph. Eur.) and calcium acetate hydrate (extra pure) were purchased from Merck KGaA (Darmstadt, Germany). Tannic acid was bought from MP Biomedicals (Irvine, CA, USA). Ethanol (99.8% denatured with IPA) was purchased from AppliChem (Darmstadt, Germany). Disodium hydrogen phosphate dihydrate (Fluka) and sodium acetate trihydrate (Riedel-de Haën) were purchased from Honeywell (Offenbach, Germany). Transglutaminase (ACTIVA_®_ WM) was kindly provided by Ajinomoto (Paris, France). All chemicals were used as received with no further purification.

### 2.2. MNP synthesis

Iron oxide nanoparticles were continuously synthesized through the precipitation of alkaline solutions of iron chloride in an aqueous medium using a micromixer set-up, as previously reported.^29^ A caterpillar micromixer (Fraunhofer IMM, Mainz, Germany) was used to mix solutions of iron chloride, sodium nitrate and sodium hydroxide. The mixture was then pumped through a 52.5 °C reaction loop with a residence time of 4 min, maintaining symmetric liquid ratios. Tannic acid was used as stabilizing agent. Then, purification of the MNP by magnetic separation was carried out to remove unreacted starting materials and to withdraw uncoated stabilizing agent.

### 2.3. Casein coatings on MNP

The casein coating process was adapted after a previously reported coating procedure for BSA.^6^ Briefly, watery MNP solution was gently mixed with the same volume of a 10 mg mL^−1^ casein solution in carbonate-buffer (50 mmol L^−1^, pH 8.5). The mixture was shaken for 16 h at 60 °C and the coated MNP were cleaned afterwards by magnetic separation with a “magnetic column” (Miltenyi Biotec, Bergisch Gladbach, Germany). The unattached casein was washed out with carbonate-buffer 5-times the sample volume and the clean sample was collected in carbonate-buffer 1/3 the sample volume.

### 2.4. Crosslinking with transglutaminase (mTG)

Crosslinking of Na-caseinate coated MNP were adapted from the procedure of Duerasch *et al.*^30^ Here, mTG was dissolved in a phosphate buffered saline (PBS) solution (pH 7.4) and an enzyme activity of 400 nkat per g Na-caseinate was used. Crosslinking was performed at 40 °C for 1 h, with a subsequent deactivation of mTG at 85 °C for 7 min.

### 2.5. Stability studies

The uncoated and coated MNP were mixed with salts and the particle size and magnetic behaviour were analysed afterwards. Solutions of NaCl, Ca(CH_3_COO)_2_, (NH_4_)_2_SO_4_ and MgSO_4_ were prepared, concentrations of 0.2 mol L^−1^, 0.1 mol L^−1^ and 0.04 mol L^−1^ were each mixed with the same volume of the sample. After 2 h and 18 h pictures were taken. DLS, DCS and MPS were performed after 18 h.

Furthermore, temperature stability was investigated by DLS measurements. Samples were stepwise heated up from 20 °C to 60 °C and cooled back to 20 °C, with DLS measurements carried out every 2 °C.

### 2.6. Determination of the encapsulation efficiency of model drug (nile red)

Encapsulation efficiency was determined with nile red (0.1 mg mL^−1^ in ethanol). To 1 mL of the coated MNP, 100 μL of the nile red solution was added. The nile red was added in the last hour of the coating process, allowing the process to continue for 1 h at 60 °C. Clean-up was done as described in section 2.3, but to determine the encapsulation efficiency (EE%), nile red was extracted with ethanol, while the particles were still adsorbed in the column, with 1/3 of the original volume. The concentration was determined by UV/Vis-spectroscopy (550 nm for nile red in ethanol) with a UV/Vis-spectrometer (Cary 50, Varian, Palo Alto, CA, USA) and single use polystyrene cuvettes (Sarstedt, Nümbrecht, Germany). The calibration curve of nile red was prepared in ethanol (8 μg mL^−1^, 5 μg mL^−1^, 4 μg mL^−1^, 3 μg mL^−1^, 2 μg mL^−1^, 1 μg mL^−1^, 0.5 μg mL^−1^, 0.25 μg mL^−1^).

### 2.7. Release study of model drug (nile red)

Samples of casein coated MNP (non-crosslinked and crosslinked) with encapsulated nile red were produced as described before but were collected from the magnetic column in PBS (pH 7.4). In this manner, 4 mL of each sample (*n* = 3) of coated MNP were produced. The release was started with the dilution of the samples with PBS (pH 7.4) to a total volume of 36 mL. The release was carried out at 37 °C and the release buffer contained 5% polysorbate 80. After certain time intervals (10 min, 30 min, 1.5 h, 3 h and 6 h) an aliquot of 1 mL was taken and run over the magnetic column. The cleaned volume was collected and the released nile red was analysed fluorometrically (ex. 554 nm, em. 638 nm). The concentration was calculated with a linear calibration of nile red in the release buffer (0.5 μg mL^−1^, 0.25 μg mL^−1^, 0.1 μg mL^−1^, 0.05 μg mL^−1^, 0.01 μg mL^−1^, 0.005 μg mL^−1^). Fluorometric analysis was carried out with a microplate reader (Spark®, Tecan, Männedorf, Switzerland) in a black 96 well plate (Thermo Scientific 9502867).

### 2.8. Iron concentration

The iron concentration *c*(Fe) of the MNP samples was determined using a phenanthroline protocol through UV/Vis-spectroscopy.^31^ A volume of 10 μL MNP was dissolved in 40 μL of HCl (37%). After complete dissolution, 450 μL ultrapure water was added. Hydroxylamine hydrochloride (10%, 50 μL) and 1,10-phenanthroline hydrochloride (0.1%, 150 μL) were added to 50 μL of this solution in a 96 well plate. After 35 min reaction time, the absorbance of the formed ferroin complexes were measured (510 nm) using a microplate reader (Spark®, Tecan, Männedorf, Switzerland). The concentration was calculated by a calibration curve (iron(ii,iii) oxide) within the concentration range *c*(Fe) = 0 to 40 mmol L^−1^. Samples were analysed in triplets.

### 2.9. Magnetic characterisation

#### 2.9.1. DC magnetization measurements

Room temperature (*T* = 295 K) DC magnetization measurements (DCM) of the MNP samples were performed using a SQUID magnetometer (MPMSXL, Quantum Design, USA). The device measures the magnetic moment *m*(*H*) of a 50 μL sample volume in the fluid state as a function of an external magnetic field *H* up to 4 × 10^6^ A m^−1^ (*B* = 5 T). The (mass) magnetization *M*(*H*) (in units A m^2^ per kg(Fe) or emu per g(Fe)) is obtained by normalizing to the total iron amount of the sample and the saturation magnetization *M*_S_ = *M*|_*B*=5 T_ was determined after subtracting a linear (paramagnetic or diamagnetic) background contribution from the sample container, water and non-magnetic sample material. An overall measurement uncertainty of about 5% with 2% contribution from the measurement device and 3% due to preparation and the iron concentration determination is estimated. We did not perform any fitting of *M*(*H*) curves using the common Langevin model because the assumptions of isotropy (no crystal anisotropy) and thermal equilibrium of this model at room temperature become invalid for diameters larger than 20 nm.

#### 2.9.2. Magnetic particle spectroscopy

In magnetic particle spectroscopy (MPS) a sinusoidal excitation field is applied to the magnetic sample and the nonlinear dynamic magnetic response of the MNP moments is detected. From the MPS spectra, the parameters *A*_3_ (amplitude of 3. harmonic) and *A*_5_/*A*_3_ (ratio of 5th to 3rd harmonics) were extracted. These depend on the MNP size distribution,^32^ magnetic anisotropy, binding state^33^ and interparticle interactions^34^ and give information about the performance of MNP in magnetic particle imaging and physical properties like aggregation state and magnetic properties. The MPS parameter *A*_5_/*A*_3_ is independent of concentration, in this case the preparation uncertainty (∼2% of pipetting) is of no importance, so this parameter can be determined with an uncertainty below 1%. As they are smaller than the symbol width they are not displayed in the corresponding graphics.

##### 2.9.2.1 Device and settings

The measurements were performed at body temperature (*T* = 37 °C) using a commercial MPS device (MPS-3, Bruker, Rheinstetten, Germany), operated at a sinusoidal excitation field amplitude *B* = 25 mT at a fixed frequency of *f*_0_ = 25 kHz. Samples of 10 μL volume filled into a PCR tubed were first measured in a fluid state and then immobilized (to suppress the rotational degrees of freedom or Brownian motion) by addition of gypsum powder into the PCR tube.

For the MPS measurements during the salt stability tests, we used our novel inline-MPS device originally developed for sensitive MNP detection under flow conditions during MNP synthesis.^35^ All measurements done with the inline-MPS were measured at *B* = 12 mT.

#### 2.9.3. AC-magnetometry (hyperthermia measurements)

All AC-magnetometry (hysteresis) measurements were carried out using a commercial AC magnetometer system (“AC Hyster™”, Nanotech Solutions S.L., Villacastín, Spain). The system is designed for characterising liquid nanoparticle samples of 40 μL. The integrated fluidic cooling system is connected to the coil of the device to prevent heat leakage from the coil operation into the sample space. Following the manufacturer's instruction, the system is limited to nanoparticle samples with *c*(Fe) of less than 10 g L^−1^ to prevent disturbing the coil system of the device. The hysteresis behaviour of the sample in response to the drive-field is measured by a pick-up coil. The area *A* enclosed by the measured hysteresis loop is then analysed and recorded in units of mJ per kg(Fe). The specific loss power of the MNP sample for the given drive field of intensity *H*_0_ and frequency *f* is then calculated based on SLP = *A* × *f*. From that, the intrinsic loss power is calculated with ILP = SLP/(*H*^2^ × *f*) which is independent of the external parameters *H* and *f*. The AC magnetometer device can provide measurements under drive-field conditions spanning a wide variety of frequencies and amplitudes. The measurements are performed in triplicates with a resulting uncertainty of about 3%. In this study, we measured the hyperthermia behaviour at a frequency of 100 kHz and an amplitude of 24 kA m^−1^.

### 2.10. Dynamic light scattering and zeta potential

For dynamic light scattering (DLS) and zeta potential (ZP) measurements a Zetasizer Ultra-Red (Malvern Panalytical ltd. Worcestershire, UK) with the ZS Xplorer (v2.3.1.4) software was used. DLS measurements (back scattering) were performed at 25 °C in DTS0012 cuvettes with water as dispersant and magnetite (*n* = 2.36) as analysed material. For ZP measurements, DTS1070 folded capillary cells were used. An equilibration time of 120 s and 300 s (temperature stability) was set. All Measurements were automatically performed in triplicates with a resulting uncertainty of around 5%.

### 2.11. Differential centrifugal sedimentation

Differential centrifugal sedimentation (DCS) measurements (CPS Instruments Inc. Measurements, Darmstadt, Germany) were conducted at 20 000 rpm (equivalent to 21 504*g*) after calibrating with a silicon dioxide standard (245 nm). A sucrose gradient ranging from 24% to 8% was used. The peak maximum and full width at half maximum (FWHM) were determined using Origin® software.

### 2.12. Transmission electron microscopy

Transmission electron microscopy (TEM) measurements were performed to determine the size and shape of the core and protein shell. Sample preparation was done by drop casting the sample on a carbon-coated copper grid. A magnetic field was applied for ∼15 min to gather the MNP. The solvent was evaporated at room temperature. The measurement was conducted using a Zeiss Libra 120 electron microscope (Zeiss, Oberkochen, Germany) operating at an acceleration voltage of 120 kV. A CCD camera was used to capture the images. The obtained images were analysed using the open-source software ImageJ (National Institutes of Health, Bethesda, MD, USA) to determine the average diameter and standard deviation of the individual nanoparticles (*N* > 1000).

### 2.13. Cell cultivation, cell viability assay and uptake

Squamous Cell Carcinoma cells (SCL-1) were cultivated in Dulbecco's Modified Eagle Medium (DMEM) low glucose (Gibco), including 10% (v/v) foetal calf serum and 1% penicillin/streptomycin. For cytotoxicity testing, 12 000 cells per well were seeded in a 96 well plate and cultivated for 24 h at 37 °C and 5% CO_2_. Samples were analysed in duplicates with a final concentration of 10% (v/v) in 100 μL per well. A 10% PBS solution was added as a control. After 72 h, the medium was removed and a Cell Counting Kit-8 (Sigma-Aldrich, Taufkirchen, Germany) performed according to manufacturer's specifications. A 10% (v/v) working solution was added to each well and incubated for 1.5 h at 37 °C. Absorbance was measured at 450 nm using a Spark Plate Reader (Tecan, Männedorf, Switzerland).

With a concentration of 36 000 cells per well, SCL-1 were seeded in an 8 well μ slide (ibidi, Gräfelfing, Germany) and cultivated for 24 h at 37 °C. Samples were added using the highest cell compatible concentration determined in the cytotoxicity assay and incubated for 24 h, 48 h and 72 h. Wheat germ agglutinin Alexa Flour 488 (Invitrogen) was used for cytoplasm staining according to manufacturer's specifications. Cells were fixed with 3.7% paraformaldehyde, washed twice and incubated with 4′,6-diamidino-2-phenylindole (Life Technologies) for nucleus staining. Images were taken using a BZ-X810 fluorescence microscope (Keyence, Neu-Isenburg, Germany). The exposure time of the red channel, for particle imaging, was established using the control well and not increased for sample imaging.

## 3 Results and discussion

### 3.1. MNP synthesis/coating and characterisation

**Fig. 2:**
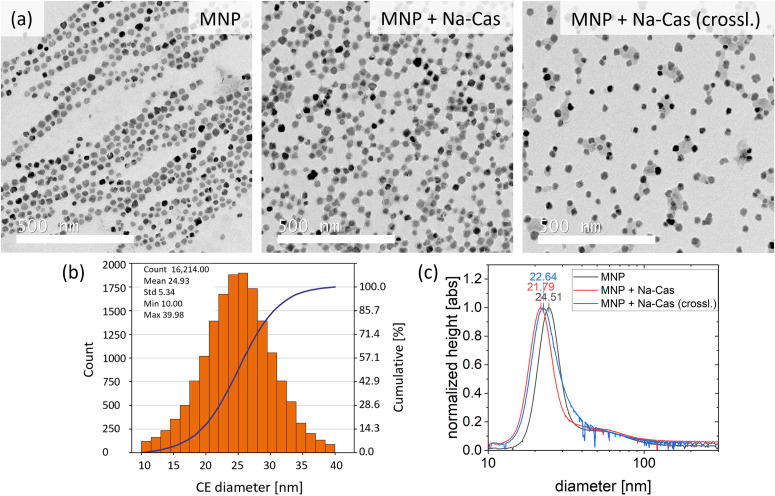
TEM pictures of (a) uncoated MNP, MNP coated with Na-Cas and MNP coated with Na-Cas enzymatically crosslinked. Scale bar 500 nm. In (b) the size distribution, derived from (a) of uncoated MNP is shown. In (c) the size distribution of uncoated and coated MNP from DCS measurements are shown.

Single core iron oxide nanoparticles were synthesized by a continuous flow micromixer synthesis. This continuous process offers a reliable and reproducible MNP production.^36^ The obtained MNP show a narrow size distribution with a mean diameter of 24.9 nm ± 5.3 nm determined by TEM and being in agreement with the hydrodynamic diameter of 24.5 nm (FWHM 8.8 nm) determined by DCS (Fig. 2). Using DCS measurements, the MNP size distribution exhibits a minimal shoulder from 40 nm to 100 nm which can be attributed to a small fraction of dimers or trimers and aggregated particles. The synthesized MNP were used for surface modification with different types of casein coatings, similar to a previously reported method for BSA coating.^6^

One sample was coated with Na-caseinate (MNP + Na-Cas) and the second sample was additionally crosslinked with transglutaminase (MNP + Na-Cas (crossl.)). All MNP were investigated using TEM and DCS, as shown in Fig. 2 and ESI Fig. 1† (TEM with lower magnetization).

Fig. 2 illustrates the TEM images of uncoated and coated MNP. The image of the uncoated MNP shows the particles arranged by an external magnetic field, however, the casein coated MNP do not display this behaviour. MNP + Na-Cas reveal a thin additional layer around the MNP, which is most likely casein. This would be in agreement with the MNP coated with BSA.^6^ By crosslinking the MNP + Na-Cas with transglutaminase, the thin layer of protein does not increase in thickness but some of the MNP are interconnected with sheet-like structures.

DCS analysis of casein coated MNP reveals an apparent smaller particle size than uncoated MNP (Fig. 2(c)). MNP + Na-Cas have a size of 21.79 nm (FWHM 8.5 nm) and MNP + Na-Cas (crossl.) 22.64 nm (FWHM 10.26 nm). As this is not in agreement with the TEM images which show the formation of a layer around the particles, the decrease in size has to be explained otherwise. One explanation could be that the DCS method is based on the particle density.^37^ If the particles have the same density, a size comparison between a known standard and an unknown sample could be made. The casein layer has a much lower density compared to the iron oxide core and therefore reduces the density of the MNP-system. This leads to a slower sedimentation and, for the same assumed density of the magnetite core as before, ultimately to a smaller particle size.

The MNP-systems were further analysed in terms of size (ESI Fig. 4†) and zeta potential (ZP, ESI Table 1†) using DLS. MNP reveal a relatively large average size and a moderate size distribution with 79 nm and a PDI of 0.27. For the casein coated MNP the z-average increased to 128 nm (PDI 0.22) for MNP + Na-Cas and to 99 nm (PDI 0.17) for MNP + Na-Cas (crossl.) compared to the uncoated MNP. The DLS measurements show significant larger particle sizes than the DCS and TEM measurements, which is due to the examination of the hydrodynamic radius. Still, a difference between the two casein coatings can be observed. MNP + Na-Cas are larger in size compared to uncoated MNP. The additional layer of casein surrounding the MNP increases the radius and can further bind water from the environment, leading to a swelling and additional increase of their hydrodynamic size. After crosslinking (MNP + Na-Cas (crossl.)), the particle size decreases by approximately 30 nm. The crosslinking with transglutaminase is inducing covalent bonds between γ-carboxamide groups of glutamine and a primary amine,^38^ bringing the protein chains closer together and reducing the possibilities of binding water. Therefore, the smaller size could originate from a tightly packed layer and less swelling due to water adsorption in the casein layer. Even though the data from TEM and DCS cannot directly be compared to the DLS measurements, the observed trends are similar. Casein coating reduces the density of the MNP-system, resulting in smaller sizes measured by DCS, while DLS analysis shows an increase in size, as would be expected.

The ZP of all samples was additionally determined (ESI Table 1†). The MNP have a negative ZP of −53.87 mV ± 2.19 mV. The high negative charge originates from the polyphenol groups of tannic acid at the surface of the MNP, which was used as stabiliser.^6^ After the coating process, the MNP + Na-Cas and MNP + Na-Cas (crossl.) reveal a moderate ZP of −36.71 mV ± 0.60 mV and −38.10 mV ± 4.62 mV, respectively. The isoelectric point of casein is approximately 4.6, therefore casein is also negatively charged at a pH of 6.7.^10^ The ZP of MNP + Na-Cas and MNP + Na-Cas (crossl.) respectively, is in the range of comparable casein particles, with a ZP of pure casein particles or micelles in the range of −20 mV to −33 mV depending on their manufacturing, the solvent and the composition^10,18^ and of reported casein MNP-systems with ZP of −22 mV to −38 mV.^23,25,26,39^ This further indicates that the coating process was successful and is completely covering the MNP, as was already observed in the TEM images.

Overall, this characterisation shows that our synthesis and coating protocol can successfully produce casein coated MNP with a narrow size distribution. In contrast, other approaches with a narrow size distribution of casein coated MNP have either a more complex coating process or a more complex MNP-system and also have smaller MNP diameter. For example, hydrophobic MNP which first need a ligand exchange of oleic acid to oligosaccharide before coating with casein (∼15 nm)^26^ and the reported MNP-polymer-casein layer-by-layer system (∼10 nm).^27^ In another reported approach, MNP are produced directly inside of casein micelles, which, in contrast to our process, leads to a wide size distribution and anisotropic MNP (50 nm to 300 nm).^25^

Analysing the MNP and the successful casein coating is the basis for the following investigation of the magnetic behaviour (3.2 and 3.3) and the stability studies (3.4).

### 3.2. Magnetic characterisation (DCM): saturation magnetization and moment estimation

**Fig. 3:**
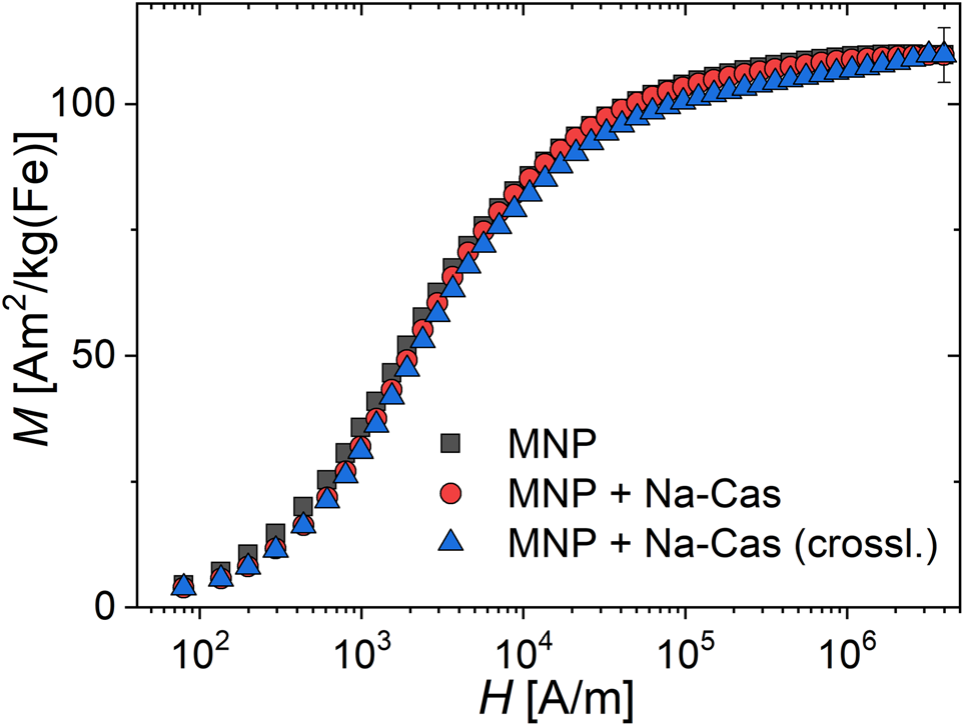
Room temperature (*T* = 295 K) DC-magnetization curves of the three MNP-systems MNP (black squares), MNP + Na-Cas (red circles), and MNP + Na-Cas (crossl.) (blue triangles). Note the logarithmic field scale for visualizing the magnetic behaviour at lower fields.

The saturation magnetization *M*_S_ of magnetic nanoparticles is a valuable magnetic parameter that indicates the quality of the crystal structure and its homogeneity achieved by the nanoparticle synthesis process, after casein coating and enzymatic crosslinking. The room temperature magnetization curves for the three systems are shown in Fig. 3, from which the saturation magnetization *M*_S_ is determined. We observe *M*_S_-values at the same value of about 109(5) A m^2^ per kg(Fe) for all systems. These values are very close to *M*_S_-values (111 to 127 A m^2^ per kg(Fe)) reported for bulk magnetite or maghemite.^40,41^ That indicates high crystallinity of the magnetite structure with a low amount of disorder achieved by our synthesis. In contrast, in the study by Bani *et al.*^39^ a *M*_S_-value of 60 A m^2^ per kg(Fe) [emu g^−1^] for uncoated and 44.86 A m^2^ per kg(Fe) [emu g^−1^] for casein coated MNP was reported, whereas we find much higher values for all our systems. The higher the *M*_S_-value, the better the performance of the MNP-system for imaging and diagnosis using magnetic techniques such as MPI or hyperthermia. As all our three systems exhibit the same *M*_S_ within the measurement uncertainty, this confirms that the coating and crosslinking of the MNP does not alter the magnetic behaviour. The observed reduced magnetization of the MNP + Na-Cas and MNP + Na-Cas (crossl.) seen at lower field strengths (*H* < 10^4^ A m^−1^) could be attributed to slightly reduced magnetic interactions between the coated MNP, reducing chain formation in the fluid samples.

### 3.3. Investigation of magnetic behaviour of casein coated MNP for potential application in MPI monitored drug delivery and hyperthermia

**Fig. 4:**
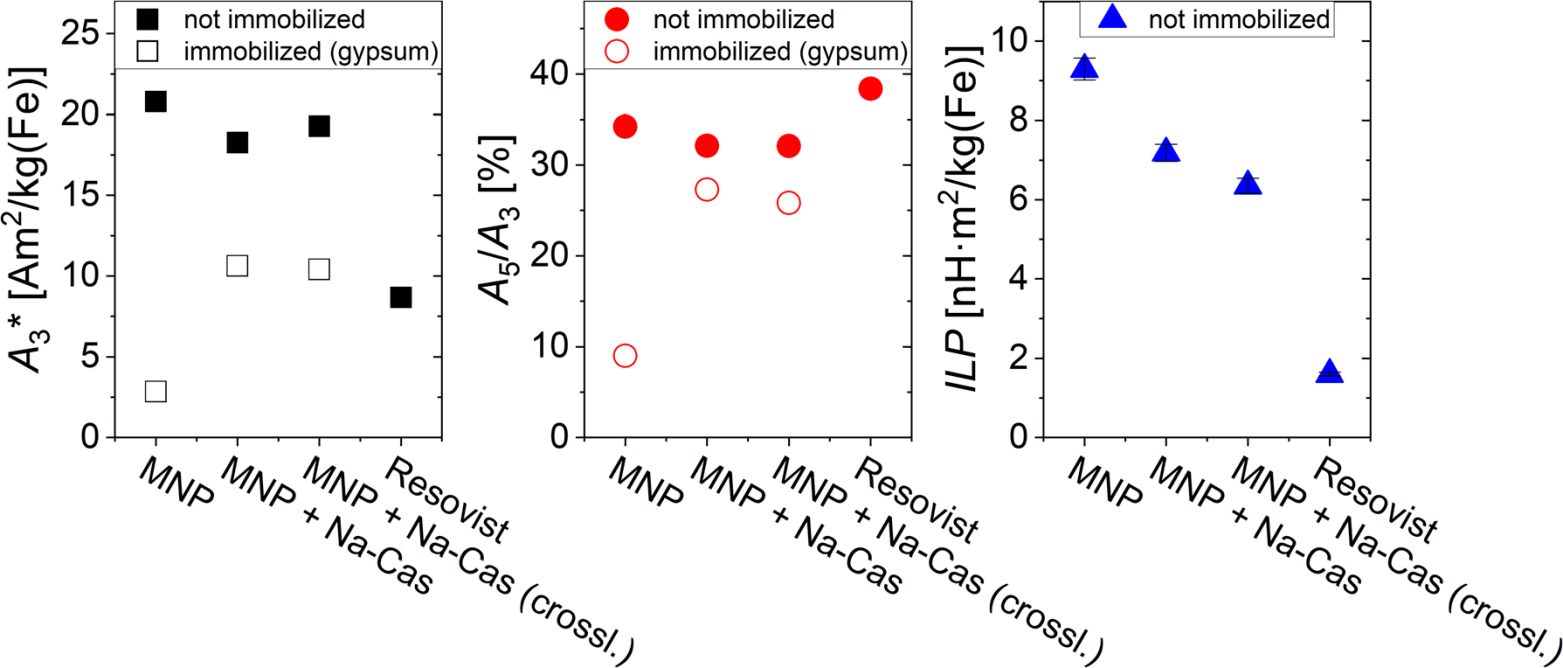
*A*_3_*, *A*_5_/*A*_3_, and ILP-values of uncoated and coated MNP and the MRI contrast agent Resovist as a reference.

The magnetic behaviour of all samples was investigated using MPS and AC-magnetometry. The applied methods are sensitive to detect minimal changes due to differences in the environment (presence of salt) or different states (in solution *versus* immobilized). In Fig. 4, the parameters *A*_3_* (MPS moment normalized to iron concentration) and *A*_5_/*A*_3_ (concentration independent) which were determined by MPS and the ILP determined through AC-magnetometry are displayed. In addition to our MNP (uncoated and coated), the MRI contrast agent Resovist is shown as a reference. MPS measurements show a decrease of *A*_3_* for coated MNP of <15%, from 20.79 A m^2^ per kg(Fe) (MNP) to 18.27 A m^2^ per kg(Fe) (MNP + Na-Cas) and 19.28 A m^2^ per kg(Fe) (MNP + Na-Cas (crossl.)). These values are more than twice as high as *A*_3_* of Resovist (8.67 A m^2^ per kg(Fe)). *A*_5_/*A*_3_ showed a slight decrease from 34.24% (MNP) to 32.14% (MNP + Na-Cas) and 32.10% (MNP + Na-Cas (crossl.)), indicating that casein coating does only marginally influence the MPI performance of the MNP. The decrease of the MPS parameters can be attributed to the increased hydrodynamic diameter of MNP with casein coating, which might cause a reduced ability of the magnetic moment of the MNP to follow the excitation magnetic field. Similar effects of reduction in MPS parameters due to hydrodynamic diameter were previously demonstrated with BSA coated MNP.^6^ The uncoated and coated MNP were immobilized and measured using MPS. The uncoated MNP displayed a strong decrease of *A*_3_* (2.84 A m^2^ per kg(Fe)) and *A*_5_/*A*_3_ of about 25% (9.01%), while *A*_5_/*A*_3_ of the coated MNP only decreased about 6%. This indicates that the immobilization affects the dynamic properties of casein coated MNP less strongly compared to the uncoated MNP. Therefore, it should be feasible to measure the magnetic behaviour or MPI of the casein coated MNP in cells or other biological matrices, where the MNP may be in an immobilized or bound state.

In addition to the MPS measurements, the ILP was determined through AC-magnetometry. As expected, the MRI contrast agent Resovist only shows a low ILP of 1.6 ± 0.05 nH m^2^ per kg(Fe), while the MNP have a higher ILP of 9.29 ± 0.28 nH m^2^ per kg(Fe) before coating and still remarkable high levels of 7.18 ± 0.22 nH m^2^ per kg(Fe) (MNP + Na-Cas) and 6.39 ± 0.19 nH m^2^ per kg(Fe) (MNP + Na-Cas (crossl.)) after coating. This high heating performance is in the range or higher of other systems and should allow efficient hyperthermia treatment.^42,43^ With increasing ILP, the same hyperthermia efficiency can be achieved with a reduced concentration of MNP. Therefore, a maximal ILP-value is desired to be able to reduce the required amount of MNP for a significant performance in magnetic hyperthermia.

This investigation of the magnetic behaviour of the casein coated MNP demonstrates their suitability to act as a hyperthermia as well as a MPI agent.

### 3.4. Stability study of uncoated and casein coated MNP

**Fig. 5:**
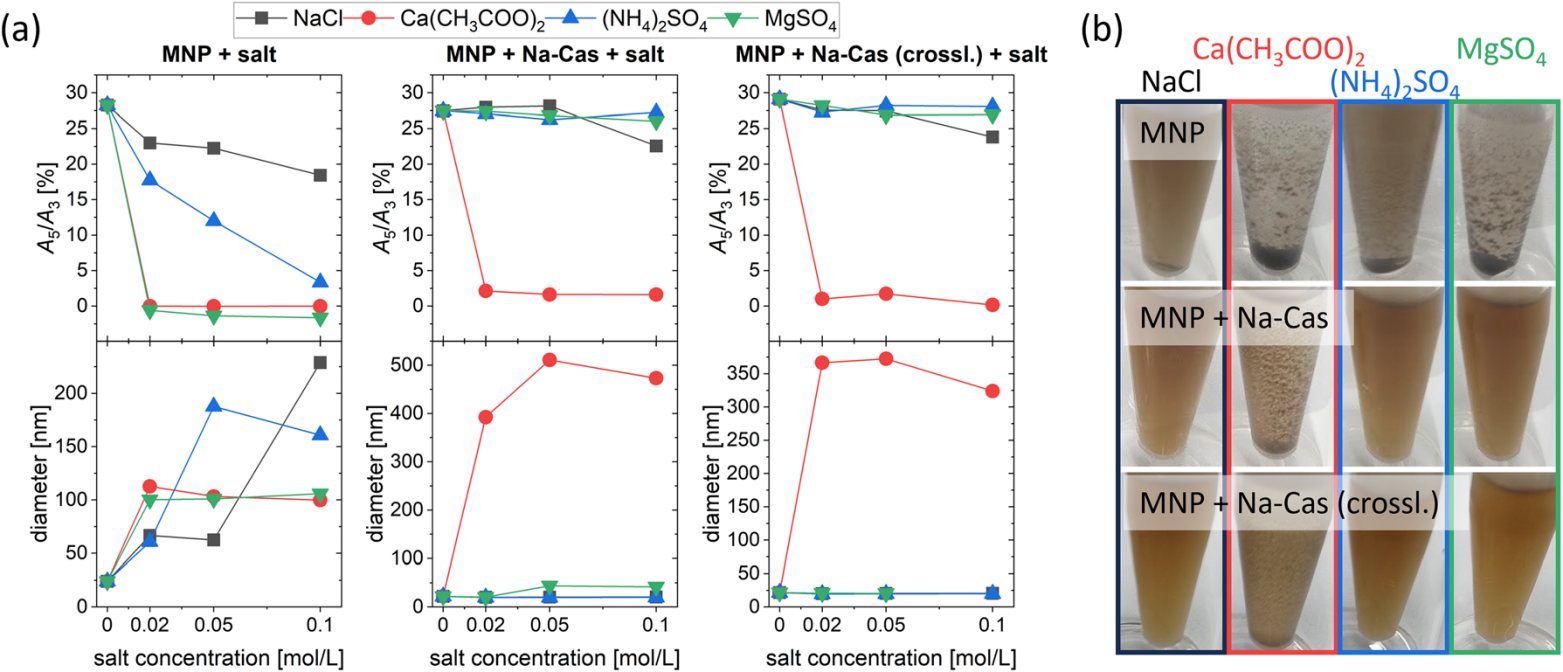
(a) *A*_5_/*A*_3_ measured with online MPS (*B* = 12 mT) and diameter measured by DCS of uncoated and coated MNP as a function of added salt concentration of NaCl, Ca(CH_3_COO)_2_, (NH_4_)_2_SO_4_ and MgSO_4_. And (b) representative images of uncoated and coated MNP at a set salt concentration (0.02 mol L^−1^) after 18 h of incubation.

A stability study was performed to compare the uncoated and coated MNP using four different salts at three different concentrations. Fig. 5 displays the MPS parameter *A*_5_/*A*_3_ and the diameter of the samples measured by DCS as a function of salt concentration. In addition, representative pictures of MNP with salt are shown at the lowest salt concentration used (0.02 mol L^−1^, 18 h). The uncoated MNP show a decrease in *A*_5_/*A*_3_ when in contact with salt (independent on kind and concentration of salt). However, for NaCl and (NH_4_)_2_SO_4_, signal loss during MPS measurement was slightly slower compared to other salts. While generally the signal drops faster with increasing salt concentrations, for NaCl the magnetic signal remains almost constant even at the highest salt concentration used (0.1 mol L^−1^) with an *A*_5_/*A*_3_ of 18.43%. Whereas for Ca(CH_3_COO)_2_ and MgSO_4_*A*_5_/*A*_3_ decreases directly towards 0 with no magnetic response left, regardless of the salt concentration. For all salts, the diameter in DCS increases, indicating the aggregation of the MNP. In agreement with *A*_5_/*A*_3_ the diameter of MNP with Ca(CH_3_COO)_2_ and MgSO_4_ increased directly towards 100 nm with increasing salt concentration without further changes. This can also be seen in Fig. 5(b), where a sedimentation and aggregate formation can be observed for all salts used. The earth alkali salt possesses a greater tendency to agglomerate the MNP than the alkali and ammonia salt.

MNP + Na-Cas and MNP + Na-Cas (crossl.) display enhanced stability compared to uncoated MNP and show almost no change in magnetic behaviour as well as in their hydrodynamic diameter. This indicates a good stabilization against both monovalent and divalent salts: NaCl, (NH_4_)_2_SO_4_ and MgSO_4_. An exception can be observed for Ca(CH_3_COO)_2_, where agglomerates and change in magnetic behaviour occur. Further, MNP + Na-Cas show a slight increase in diameter (around 43 nm) after addition of MgSO_4_ (0.05 mol L^−1^ and 0.1 mol L^−1^) which does not seem to influence the magnetic behaviour, since *A*_5_/*A*_3_ is stable at around 27%. In the DCS plot (ESI Fig. 3†), the main peak of MNP + Na-Cas did not shift but a second lower peak at around 43 nm appeared, indicating that just a small fraction of MNP + Na-Cas are aggregated and therefore the magnetic behaviour remains. Even though the casein coating was stabilizing the MNP in most of the tested salts and concentrations, they are not stable in Ca(CH_3_COO)_2_ solutions at any concentration. Casein possesses a very strong affinity and specific binding sites for Ca^2+^ which are important for the formation of natural casein micelles.^10^ In this case, the binding sites cause an agglomeration of the coated MNP. For a possible application, further studies must be done, concerning the accessible calcium in the medium.^44^ These stability studies were further confirmed by DLS (ESI Fig. 4†).

Casein is known to be temperature responsive,^22^ a promising property for drug delivery or even in combination with hyperthermia applications. The temperature dependent change of the z-average and PDI of MNP, MNP + Na-Cas and MNP + Na-Cas (crossl.) was recorded but shows no or only little effect (ESI Fig. 5†). Since the temperature change in size for casein was mainly investigated for casein micelles or aggregates,^15^ it is possible that the small layer on the MNP is simply too thin to show an effect. However, the casein coating clearly leads to a good thermal stability of the nanohybrid.

### 3.5. Encapsulation efficiency and release of nile red

Encapsulation efficiency is an important factor for potential drug delivery applications and was demonstrated with nile red (NR) as a hydrophobic model drug. After the washing step, the encapsulated nile red was extracted from the MNP (attached at the magnetic column) and the amount was determined with UV/Vis-spectroscopy. Percentage encapsulation efficiencies EE% were calculated by EE% = (NR_NP_/NR_total_) × 100 and are given in Table 1. The data in Table 1 shows that nile red can be encapsulated in the coated MNP (most likely located in the casein layer). Further, the MNP + Na-Cas (crossl.) sample can encapsulate approximately 23% more nile red. This could be due to a more stable casein layer. By crosslinking the casein layer, the already attached caseins are covalently bond to each other and cannot easily be rinsed of in the washing step. Higher amounts of casein could potentially also encapsulate more nile red. As it was seen in Fig. 2(a), more of the casein is located between the particles for MNP + Na-Cas (crossl.), forming sheet-like structures. These structures, consisting of casein, are not only connecting the MNP, they can also encapsulate nile red and increase encapsulation efficiency. By considering the iron concentration (and MNP) during the washing step, even higher encapsulation efficiency was achieved. Since the loss of iron (and MNP) between MNP + Na-Cas and MNP + Na-Cas (crossl.) was similar, while the EE% increased comparably.

**Table 1:** Encapsulation efficiencies (EE%) of nile red in the casein coated MNP, in direct relation to the used amount of nile red and in relation with the individual iron concentration

	Total EE%	EE% in relation to *c*(Fe)
MNP + Na-Cas	27.1% ± 4.7%	36.2% ± 6.3%
MNP + Na-Cas (crossl.)	50.0% ± 1.5%	74.7% ± 2.3%

Here, a straightforward, passive way of encapsulating nile red in the casein coated MNP was chosen. The encapsulation was achieved by diffusion of nile red and the resulting equilibrium between the coated MNP and the solution (with unbound casein and ethanol as solubiliser). Higher levels of encapsulation could be achieved by linking a greater amount of casein to the MNP surface, for example using a layer-by-layer approach.

**Fig. 6:**
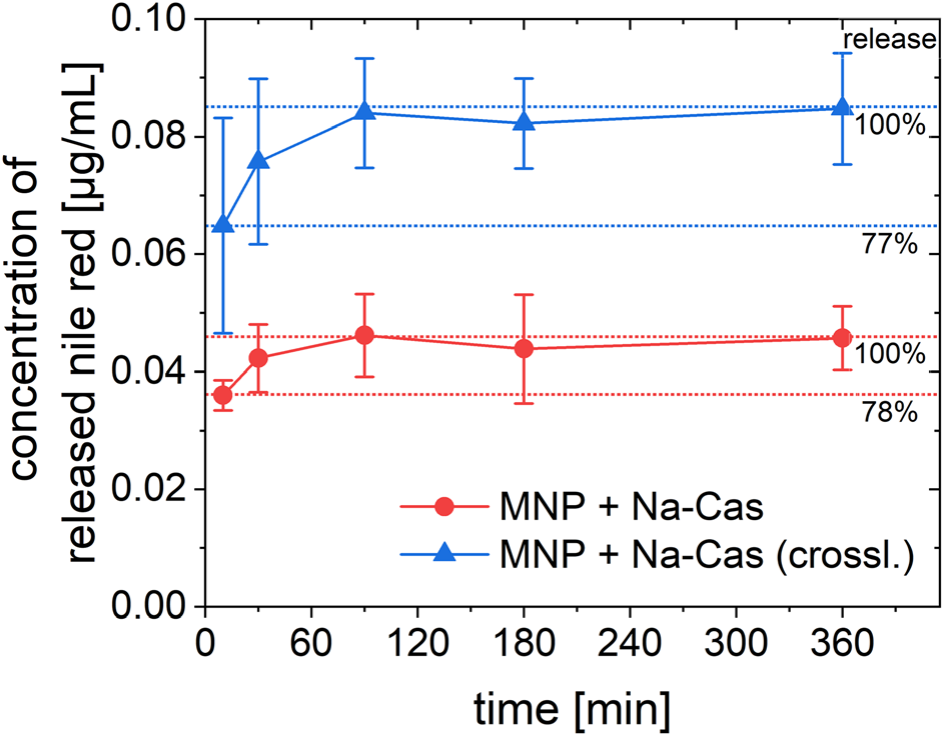
Release of encapsulated nile red over 6 h from MNP + Na-Cas and MNP + Na-Cas (crossl.) in PBS (pH 7.4) with 5% polysorbate 80 at 37 °C (MNP + Na-Cas: *n* = 3, MNP + Na-Cas (crossl.) *n* = 2).

In addition to the encapsulation efficiency, the release of the encapsulated nile red was investigated. For the release of the hydrophobic nile red, a solubiliser (5% polysorbate 80) was added to the release buffer (PBS, pH 7.4). Because of the poor water solubility of nile red, a solubiliser is needed to perform a release in aqueous medium.^45^ In fact, we could not observe any release of nile red in PBS without the addition of polysorbate. Fig. 6 shows the results of the release of nile red. For both samples the release reached a plateau after 90 min with virtually no change thereafter. After 10 min, 78% (MNP + Na-Cas) and 77% (MNP + Na-Cas (crossl.)) of the total release amount are already released, which represents a very rapid release. The MNP + Na-Cas (crossl.) released nearly twice (1.85) as much nile red compared to the MNP + Na-Cas sample, which corresponds well with the different encapsulation rates. It should be noted that a release with a solubiliser is not necessarily a direct determination of the released amount from the particles. The solubiliser can interact with the particles, leading to interpenetration and a change in the particle composition or even to degradation, but always in a substantial change in the release kinetics.^46^ Here, the release study was performed to show, that a hydrophobic drug can be released from the particles in an aqueous medium with the support of a solubiliser, as it may occur in the cytoplasm or lipid membranes of the target cells. Since nile red was only released in aqueous medium with a solubiliser present, leakage of the drug, for example during storage can be excluded.

By demonstrating that a hydrophobic model drug can be encapsulated within the casein coated MNP, an important step has been taken towards the use of these particles as theranostic agents. Many anti-cancer drugs such as doxorubicin or paclitaxel are poorly water soluble, but potentially their delivery can be improved with our casein coating, while still being able to use the MNPs for imaging purposes. The release study of the model drug (nile red) shows a rapid release for both samples, but also was only possible with the use of a solubiliser. It can be assumed that a hydrophobic drug is only released if a solubiliser is present, for example after uptake into a cell by lipophilic components.^45^ The fluorescence properties of the utilized model drug nile red also allow to investigate cellular uptake and localisation inside the cell.

### 3.6. Cell viability and uptake

We could already show the non-toxic and biocompatibility of our MNP.^47^ To investigate the cell viability of the MNP with casein coating, SCL-1 cells were cultivated for 24 h and then exposed to MNP + Na-Cas and MNP + Na-Cas (crossl.). The viability was examined with a CCK-8 assay after 72 h of exposure. No severe negative effects of the coated MNP on the SCL-1 were observed (ESI Fig. 7†). Furthermore, cell viability was not significantly affected by MNP concentration in the concentration range of 2.5 μg mL^−1^ to 20 μg mL^−1^, suggesting that casein coated MNP are a potentially non-toxic delivery system. This is in agreement with other studies which investigated the cytotoxicity for different MNP casein systems for L-929 fibroblasts^25^ and mouse derived macrophage RAW264.7.^26,48^ All studies showed no cytotoxic effect for casein coated MNP. Moreover, one study even claims that at a concentration of 200 μg mL^−1^ the cell viability remained at roughly 90%.^26,48^ The effect of the encapsulated nile red on the cell viability was additionally examined and no significant change was observed.

**Fig. 7:**
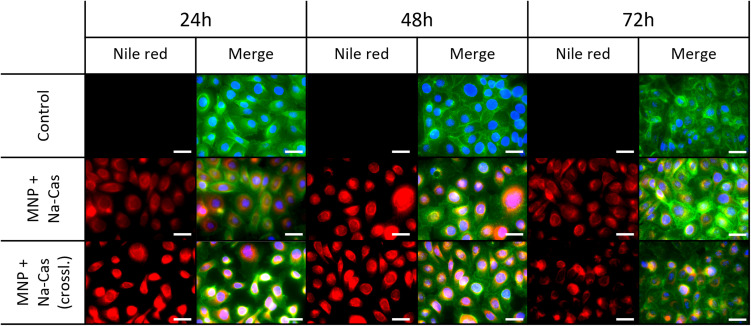
Uptake of MNP + Na-Cas and MNP + Na-Cas (crossl.) in SCL-1 after 24 h, 48 h and 72 h. Scale bar 40 μm.

Since nile red (and casein coated MNP itself) poses no harm, the cellular uptake was investigated *via* fluorescence microscopy. From the results shown in Fig. 7, a perinuclear accumulation of the nile red fluorescence signal can be observed. This points to a cellular internalization of MNP + Na-Cas and MNP + Na-Cas (crossl.) *via* endocytosis or direct cellular entry routes such as translocation or lipid fusion.^49^ It is positive, that after 24 h a strong signal of nile red already can be seen, which could indicate an uptake process.

## 4 Conclusions

Casein coated MNP have been demonstrated to perform as promising nanohybrid for theranostic application in physiological environment. The casein coating has little effect on the magnetic behaviour of the MNP. Furthermore, the colloidal stability of the coated particles against different salts with increasing concentrations was drastically improved, the additional casein layer also stabilizes the magnetic behaviour against dynamic signal losses in the presence of salts as well as in an immobilized state. A hydrophobic model drug was encapsulated efficiently in the casein layer to demonstrate the drug delivery function of the nanohybrid system. This drug carrier layer was further improved and stabilized by enzymatically crosslinking of the casein with transglutaminase. The proved non-toxicity of the carrier system itself is a crucial requirement of a save nanotheranostic system.

The successful cellular uptake in tumour cells as well as the localisation of the model drug in close vicinity to the nucleus indicate an accumulation at the site of action for a large variety of anti-cancer drugs.

We conclude that our casein coated MNP-system with its drug encapsulation capability, its physiological stability and good cell tolerance and its high magnetic performance combines all functionalities to become the basic constituent of a powerful magnetic theranostic system. For future perspectives to paving the way for a new theranostic agent into clinical application, further studies on drug encapsulation, such as the transport mechanism and degradation of the particles, are planned for ongoing studies.

## Data availability

The data supporting this article have been included as part of the ESI.†

## Author contributions

C. W. and N. M. performed the synthesis and physicochemical characterization of the samples; E. H. and V. G. planned and carried out *in vitro* cell studies. C. W., N. M., E. H., R. B., F. W. wrote the manuscript; R. B., F. W. reviewed and edited the manuscript. Work was designed and supervised by R. B. (synthesis, characterization) and F. W. (magnetic measurements); project administration, R. B. and F. W.; funding acquisition, R. B. and F. W. All authors have read and agreed to the published version of the manuscript.

## Conflicts of interest

There are no conflicts to declare.

## Supplementary Material

RA-014-D4RA02626H-s001
