# Supplementary material for: Design and characterisation of casein coated and drug loaded magnetic nanoparticles for theranostic applications
Source: RSC Adv. 2024 Aug 20;14(36):26388–99. doi: 10.1039/d4ra02626h (PMC11334153; doi:10.1039/d4ra02626h)
Supplement: RA-014-D4RA02626H-s001 [file RA-014-D4RA02626H-s001.pdf]

Supplementary Information (SI) for RSC Advances.
This journal is © The Royal Society of Chemistry 2024

Supporting information:
Design and Characterisation of casein coated and drug loaded magnetic nanoparticles for theranostic applications

Christina Wenck [a†], Nils Meier [a†], Eilien Heinrich [a], Verena Grützner [a], Frank Wiekhorst[b] and Regina Bleul[a]

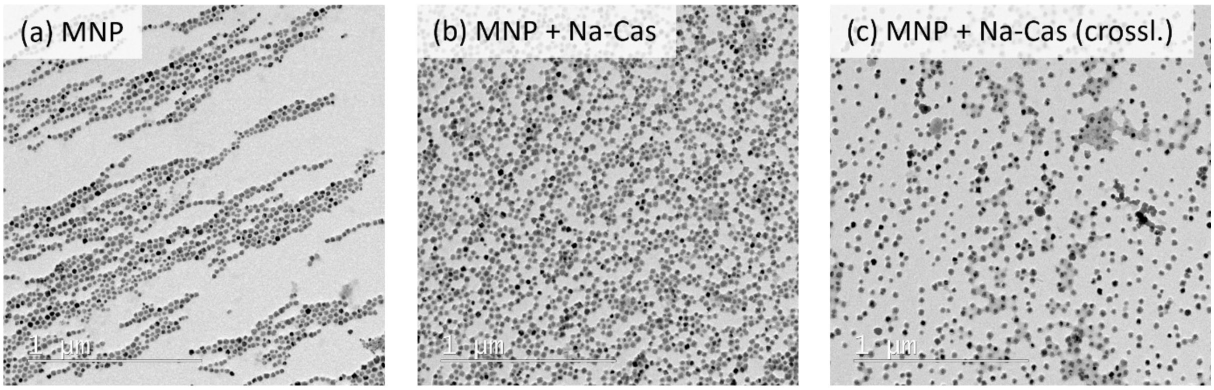

Figure 1: TEM pictures of (a) uncoated MNP, (b) MNP coated with Na-Cas and (c) MNP coated with Na-Cas enzymatically crosslinked. Scale bar 1 µm.

Table 1: Zeta potential of MNP, MNP+Na-Cas and MNP+Na-Cas (crossl.).

Sample	Zeta Potential [mV] (pH ~6.7)
MNP	-53.87 ± 2.19
MNP + Na-Cas	-36.71 ± 0.60
MNP + Na-Cas (crossl.)	-38.10 ± 4.62

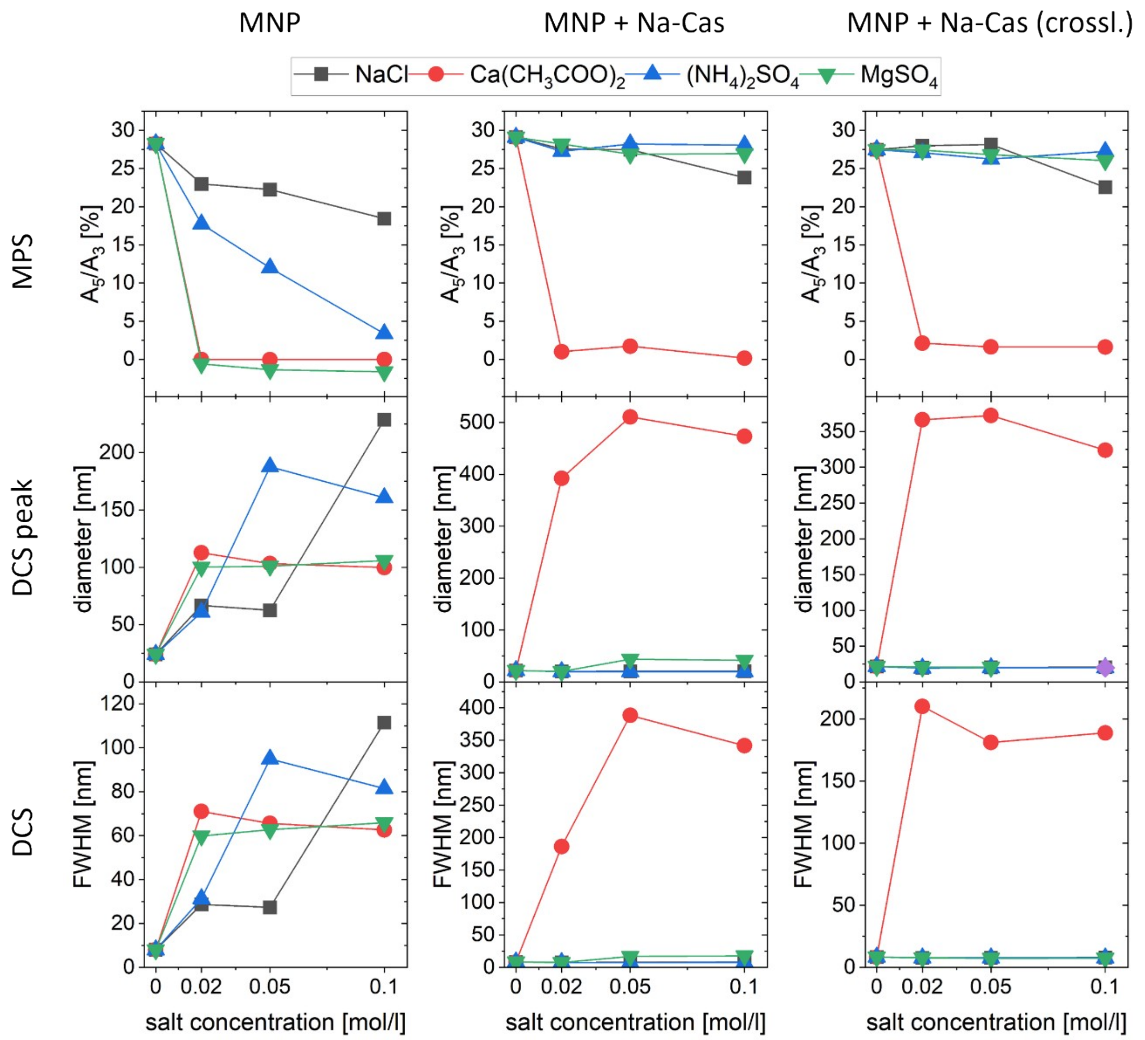

Figure 2: A_5/A_3 measured with online MPS and diameter and FWHM measured by DCS of MNP, MNP+Na-Cas and MNP+Na-Cas (crossl.) as a function of added salt concentration of NaCl, $Ca(CH_3COO)_2$, $(NH_4)_2SO_4$ and $MgSO_4$. The MPS data and DCS diameter are already shown in main article, but for comparison with FWHM they are displayed again.

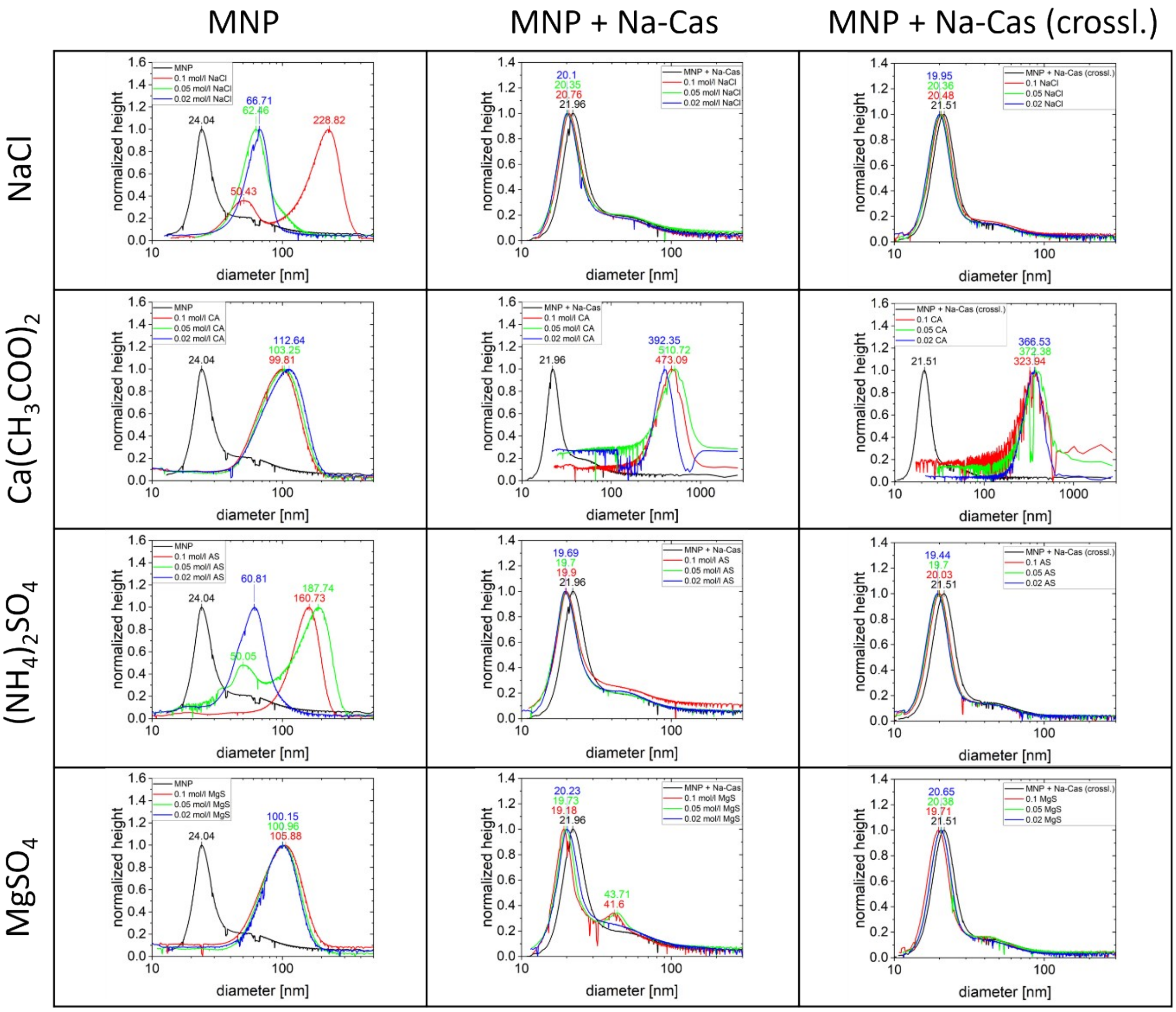

Figure 3: DCS measurements of MNP, MNP+Na-Cas and MNP+Na-Cas (crossl.) with salts NaCl, $Ca(CH_3COO)_2$, $(NH_4)_2SO_4$ and $MgSO_4$ at different concentrations: 0 mol/l (black), 0.1 mol/l (red), 0.05 mol/l (green) and 0.02 mol/l (blue).

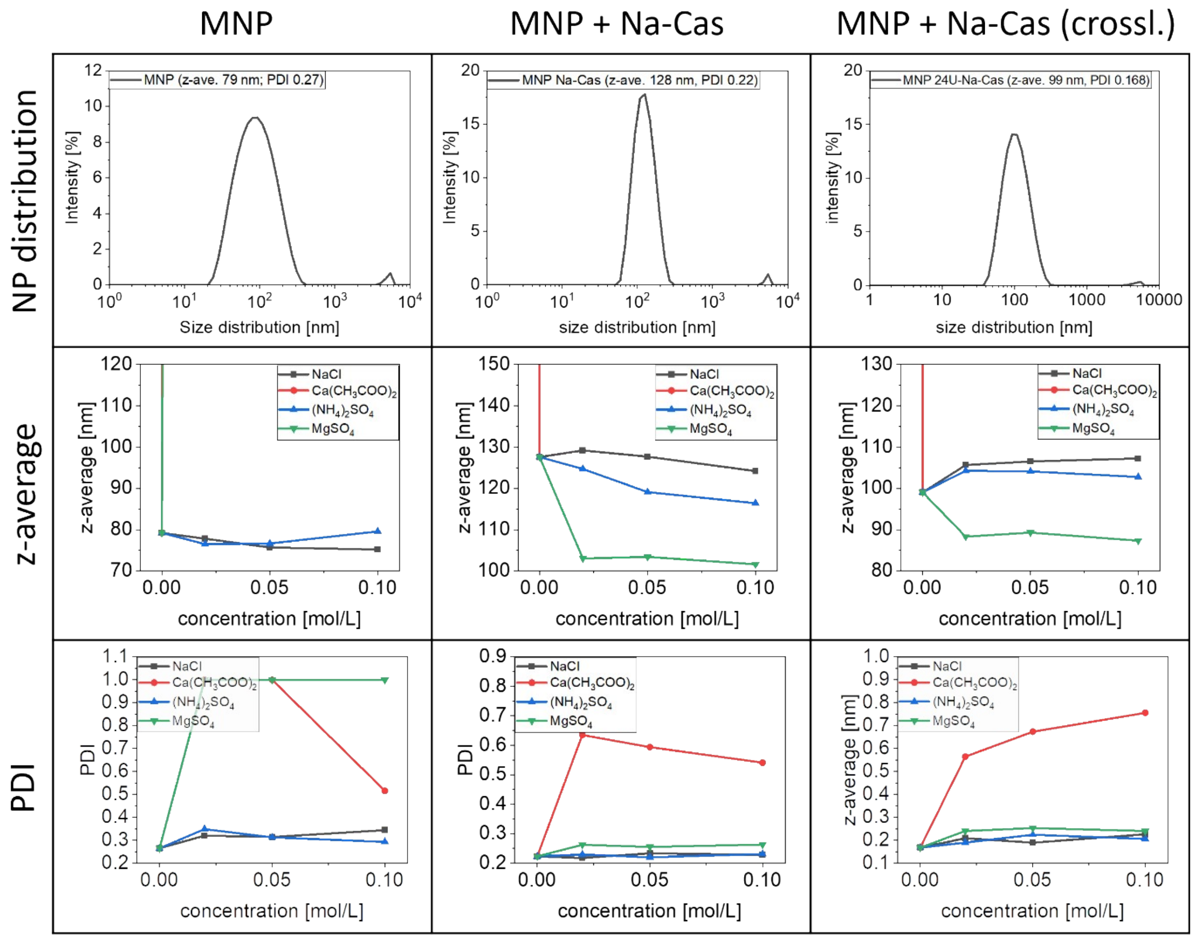

Figure 4: Z-average and PDI of MNP, MNP+Na-Cas and MNP+Na-Cas (crossl.) as a function of added salt concentration of NaCl, $Ca(CH_3COO)_2$, $(NH_4)_2SO_4$ and $MgSO_4$.

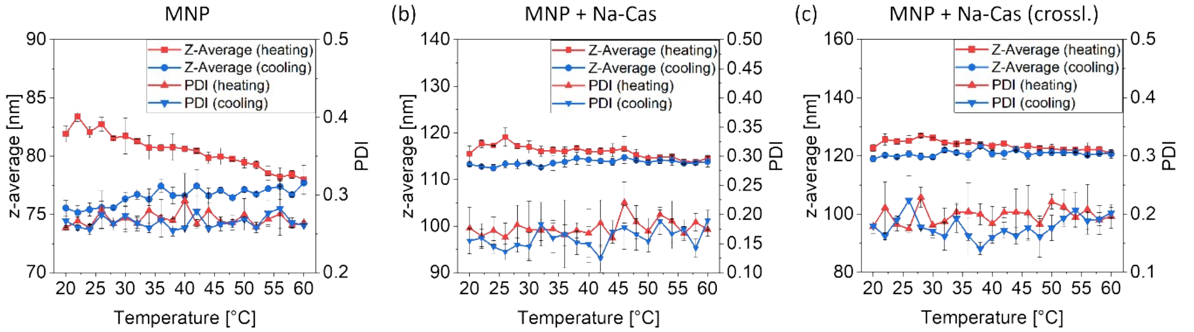

Figure 5: Z-average and PDI of MNP, MNP+Na-Cas and MNP+Na-Cas (crossl.) over a temperature change from 20 °C to 60 °C (heating) and back to 20 °C (cooling).

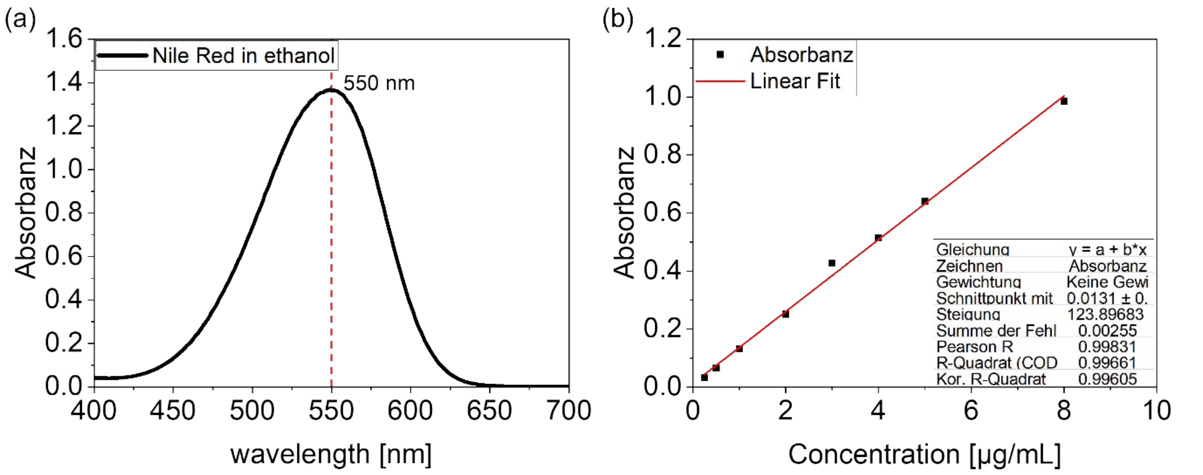

Figure 6 UV/Vis analysis of nile red, (a)Spectrum of nile red in ethanol and (b) linear calibration of nile red in ethanol for determination of encapsulation efficancy.

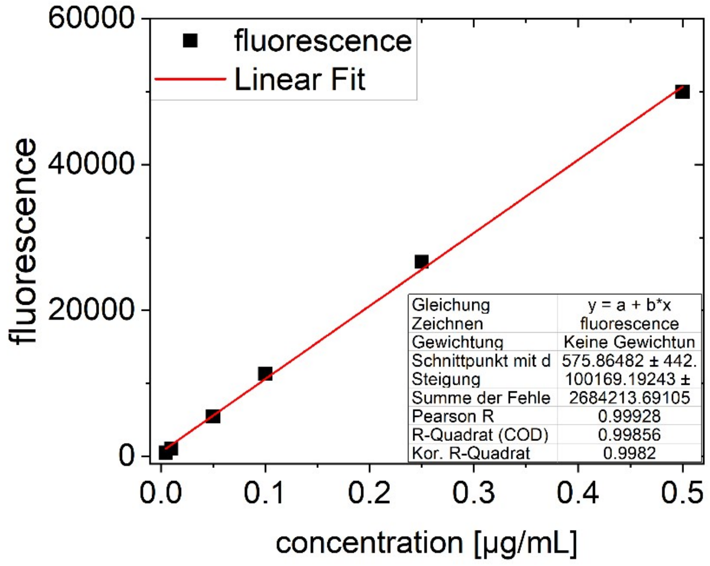

Figure 7: Fluorometric calibration curve of nile red in PBS (pH 7.4, 5% Polysorbat 80).

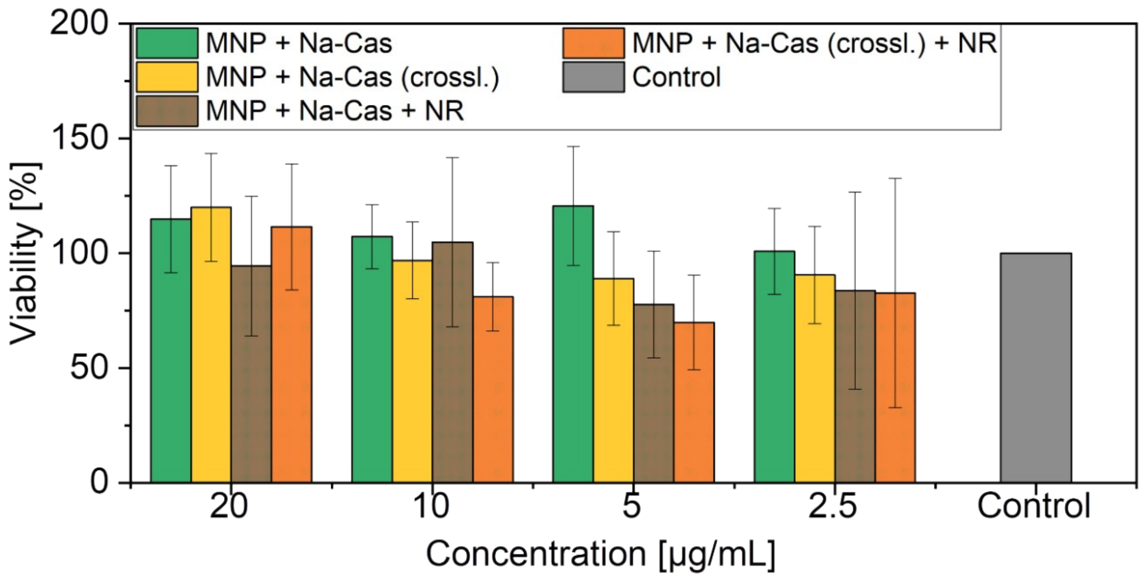

Figure 8 Viability of Squamous Cell Carcinoma (SCL-1) cells determined using a CCK-8 assay (450 nm) after 72 h of exposure to casein coated MNP (MNP+Na-Cas and MNP+Na-Cas (crossl.)) in different concentrations. Error bars represent standard deviations (n=3). NR stands for nile red.
